# Grafting of Skin Taken from Fowls, with Report of a Case

**Published:** 1888-12

**Authors:** B. F. Rogers

**Affiliations:** Passed Assistant Surgeon, United States Navy


### GRAFTING OF SKIN TAKEN FROM FOWLS, WITH REPORT OF A CASE.

BY B. F. ROGERS, M. D.,
Passed Assistant Surgeon, United States Navy.

David Williams, 2d C. F., æt. 38, native of Wales, color, white, presented himself at sick-bay for treatment on board the *Alliance*, South Atlantic station, on the evening of April 15, 1888. He said he had injured his "seat."

On examining the patient I found a patch of skin on the left buttock gangrenous. This was about six by four inches in area, surface dry, hard, black in color, as if covered with a coating of coal tar, void of sensation, and emiited a putrid odor from its sloughing margins where separation from the inflamed surrounding parts was in progress. As to the origin or cause of this state of affairs he claimed to know nothing. He was under the influence of alcoholic stimulants, indulged in for two days previous to his presenting himself for treatment. During the time mentioned he was running the engines of the steam cutter.

From others in the cutter with him I learned that he probably sat in a pan containing a solution of caustic potash. This occurred the first day of his tour of duty in the cutter, and rather than forego the opportunity to indulge his thirst for rum he bore the pain and discomfort his wound occasioned him. The patient was bathed, placed in a cot, and the wound dressed with a moist, carbolated dressing. By May 1 the slough was completely removed, leaving the substance of the gluteal muscles bare for a space indicated above. The dressing now employed was cosmoline, oiled-silk protection, and a moist carbolated compress over all.

Skin-grafting was done from May 4, the supply being taken from the patient's arms. The abundant discharges from the granulating surface, the difficulty of retaining the grafts in place, and the injury the patient unwittingly did to the wound at night while asleep by lying on it, rendered most of this work useless. May 8, grafts taken from under the wing of a young fowl, as recommended by Dr. Redard, mentioned in *Medical Record* of March 10, 1888, were placed on the granulations, and thereafter repeated as occasion required.

June 9, the surface had healed over, leaving only a space of one by one and a half inches open. On this date the patient was transferred to the British hospital at Montevideo, owing to the sailing of the ship for Punta Arenas, Chili. The condition of the patient rendered it advisable to do this. He proved a refractory patient, defied the hospital authorities, jumped the wall, and went on a continuous spree. Under these circumstances it is not to be wondered at that when the ship returned to Montevideo August 10, and received Williams on board again, his wound was in much the same condition as when I last saw him. Carbolated water dressings were again applied to reduce inflammation, and in a few days skin-grafting was resumed, fowls furnishing the supply, as before. September 14, the patient was discharged to duty.

The wound was then healed. The cicatrix is soft and pliable, and though the wound is much reduced in size by contraction, there is little of the character of a cicatrix following a deep and extensive burn about it.

